# Identifying the superior surgical procedure for endometrial cancer: A protocol of network meta-analysis

**DOI:** 10.1097/MD.0000000000016855

**Published:** 2019-08-16

**Authors:** Min Yin, Yitong Cai, Li Zhou

**Affiliations:** a The First Hospital of Lanzhou University; b School of Nursing, Lanzhou University; c Gansu Provincial Maternity and Child-care Hospital, Lanzhou, Gansu, China.

**Keywords:** endometrial cancer, network meta-analysis, randomized controlled trials, surgical

## Abstract

**Background:**

Endometrial cancer (EC) is one of the most common gynecologic tumors, with a high incidence in developed countries. Although the overall prognosis is good, some women have invasive tumors, the risk of recurrence, and death is high. The common surgical methods used in EC are total-abdominal hysterectomy (TAH), total-vaginal hysterectomy (TVH), laparoscopic-assisted vaginal hysterectomy (LAVH), and total-laparoscopic hysterectomy (TLH) including both conventional and robotically assisted.

**Methods:**

The literature search was performed in The Cochrane Central Register of Controlled Trials, PubMed, Web of Science, and Embase. The randomized controlled trials (RCTs) will be included. The search date is until June 2019. The risk of bias of included RCTs was assessed by 2 investigators according to the Cochrane Collaboration's tool. Network meta-analysis will be conducted by R software.

**Results:**

This study is ongoing and the results will be submitted to a peer-reviewed journal for publication.

**Conclusion:**

This network meta-analysis will provide clinical staff with current and reliable information on the best surgical approach for EC. Ethical approval is not applicable, since this is a network mate-analysis based on published articles. The protocol has been registered on PROSPERO under the number CRD42019128094.

## 1 Introduction

Endometrial cancer (EC) is one of the most common gynecologic tumors and the fourth most common cancer in the world, it ranks 14th among women in cancer mortality, and the incidence of EC in developed countries is higher than that in developing countries.^[1,2,3]^ In some countries in North America and Europe, EC has become the 3rd most common disease in women with cancer, and the incidence is 10 times higher than in developing countries.^[4]^ EC accounts for 95% of all cancer of the uterine corpus.^[5]^ According to statistics, nearly 150,000 women worldwide become new cases every year, and about 40,000 women die of this cancer.^[6]^ EC increased at a rate of 1% per year in people under age 50,^[7]^ postmenopausal women have a higher risk of EC, and about 90% of patients are older than 50 years old.^[4]^ In addition to obesity, diabetes, and hypertension, exposure to nonresistant estrogen are also important risk factors for EC.^[8,9]^ Overall, most ECs are type 1 adenocarcinoma, usually, with a better prognosis,^[10]^ 5-year survival rate is about 75%,^[11]^ early bleeding symptoms can be diagnosed immediately.^[12]^ Although EC has a good overall prognosis, some women have invasive tumors and have a high risk of recurrence and death.^[13]^

Regardless of surgical staging,^[14]^ the golden standard treatment strategy for EC is hysterectomy and bilateral salpingo-oophorectomy,^[15,16]^ at present, the common surgical methods used in EC are: total-abdominal hysterectomy (TAH), total-vaginal hysterectomy (TVH), laparoscopic-assisted vaginal hysterectomy (LAVH), and total-laparoscopic hysterectomy (TLH) including both conventional and robotically assisted. Traditionally, almost all ECs were performed by TAH. The treatment framework for EC has undergone major changes during these years, and traditional treatment models are challenged by new treatments.^[13]^ Now, many operations can be performed by minimally invasive techniques. In the past 10 years, TLH has been used more and more in patients with EC. Many studies have shown that TLH is an effective and safe option, TLH can reduce the amount of bleeding during surgery and shorten the recovery time of patients, but doctors often need 10 to 200 laparoscopic surgery experiences to achieve a stable state, and the operation time is relatively long.^[17,18,19]^ TAH was associated with an increased risk of perioperative complications compared with other surgical procedures, but it is simpler, faster.^[20,21]^ Since its publication in 1989, LAVH has been increasingly used in clinical practice and is currently considered a safe and viable technology for the treatment of benign uterine diseases, it has been recommended as an alternative to TAH for early EC.^[22,23]^ TVH had beginnings in 1507,^[24]^ in the next half century, TAH began to be used for other indications including EC,^[25]^ although new techniques for hysterectomy are emerging, vaginal hysterectomy remains the safest and most cost-effective method and is supported by numerous organizations, including the American College of Obstetricians and Gynecologists.^[26]^ With the improvement of vision and flexibility, robotic minimally invasive surgery technology has been developed, which breaks through the limitations of traditional laparoscopic surgery.^[27,28]^ It has been used effectively for benign hysterectomies, but it often comes with higher costs.^[29,30]^

In the last decade, network meta-analysis (NMA) has been introduced.^[31]^ A good NMA of randomized controlled trials (RCTs) is considered the best quality evidence for providing valid information for practice,^[32,33,34]^ and it is also the main source of key information for scientific researchers.^[35,36]^ In this study, we will use NMA to determine the best surgical approach for EC.

## 2 Methods and analysis

### 2.1 Study registration

This protocol has been registered on the international prospective register of a systematic review (PROSPERO) (https://www.crd.york.ac.uk/PROSPERO/#myprospero), and the registration number is CRD42019128094.

### 2.2 Study inclusion and exclusion criteria

#### 2.2.1 Types of studies

Any RCTs will be included, and only English literature is included.

#### 2.2.2 Types of participants

Patients diagnosed with EC and intervention group receiving any type of 5 surgical procedures will be included. Advanced patients with EC were excluded.

#### 2.2.3 Types of interventions

We included studies that performed a TAH, TVH, LAVH, or TLH including both conventional and robotically assisted.

#### 2.2.4 Types of outcome measures

Main outcomes: overall survival, recurrence-free survival, operative time, bowel injury

Additional outcomes: wound infection, blood transfusion required, cost, urethral injury

### 2.3 Search strategy

#### 2.3.1 Electronic searches

The literature search was performed in The Cochrane Central Register of Controlled Trials, PubMed, Web of Science, and Embase. The last search was performed on June 20, 2019, and still keeps updating.

#### 2.3.2 Other resources

We searched the reference list of relevant publications, abstracts of scientific meetings, and list of included studies and contacted experts in the field to identify further reports.

#### 2.3.3 Search strategies

All databases will be based on the MeSH and text word search and will be adjusted according to the specific database, take PubMed as an example, the search strategy is shown in Table 1

**Table 1:**
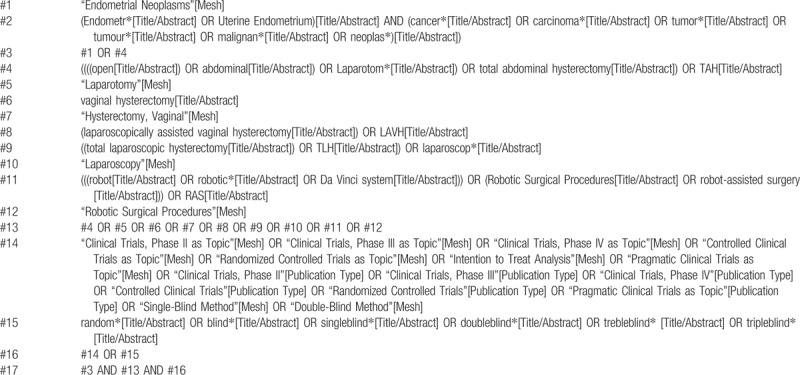
Searching strategy in PubMed.

### 2.4 Data collection and analysis

#### 2.4.1 Literature screening

All search results are imported into EndNote X8 literature management software, 2 reviewers (MY, YTC) will screen the titles and abstracts of literature independently, then read the full text to assess literature according to the inclusion and exclusion criteria, any disagreements will be resolved by a 3rd reviewer (LZ).

#### 2.4.2 Data extraction

To avoid bias, 2 reviewers (MY, YTC) independently extracted data using the same data recording form, which includes the following contents:

- General characteristics of included studies: title, name of the 1st author, publication time, study period.
- Detail of participants: gender, age, country, body mass index.
- Information about study design: total sample size, allocation sequence concealment, blinding.
- Intervention-related characteristics: name of experimental or control interventions.
- Outcomes: main outcomes and additional outcomes.

### 2.5 Study quality assessment

The risk of bias of included RCTs was assessed by 2 reviewers (MY, YTC) according to the Cochrane Collaboration's tool. This tool included 6 items: random sequence generation, allocation concealment, blinding of participants and personnel, blinding of outcome assessment, incomplete outcome data, selective reporting, and anything else ideally prespecified. In the assessment, a judgment of “yes,” “no,” or “unclear,” each domain was assigned to designate, respectively, a low, high, or unclear risk of bias. Any disagreement between the reviewers on the risk of bias will be resolved by discussion and, if necessary, by consulting a 3rd reviewer (LZ).

### 2.6 Statistical analysis

#### 2.6.1 Data synthesis

Statistical analyses will be performed using Stata 15.0 and R (version 3.4.1; R Foundation for Statistical Computing, Vienna, Austria) software. For continuous variables, weighted mean differences with 95% confidence interval (CI) will be indicated, and for dichotomous variables, risk ratios with 95% CI will be used. The NMA will be conducted in a Bayesian framework. Data analysis will be performed using R software. The result of direct comparisons will be acquired through the traditional meta-analysis. If the available data are not suitable for synthesis, we will perform a narrative review and summarize the evidences.

#### 2.6.2 Assessment of heterogeneity

We can reflect the feasibility of meta-analysis by evaluating the heterogeneity of the included studies. According to the guideline of Cochrane Handbook, heterogeneity between RCTs can be quantified using *I*-square (*I* ^2^) values, if *I* ^2^ > 50%, significant heterogeneity is considered, then a subgroup analysis is needed to determine the source of heterogeneity. If there is missing data in the included study, we will contact the author by email or phone to get the missing data.

#### 2.6.3 Subgroup analysis

If the evidence is sufficient, we will conduct a subgroup analysis to determine the difference between normal and overweight, over 60 years old and <60 years old, etc.

#### 2.6.4 Sensitivity analysis

Sensitivity analysis is used to assess the stability and reliability of the results, and explore the sources of heterogeneity, we can use random effects models or fixed effect models, relative risk, or odds ratio changes to assess if there is a change in meta-analysis results. If the meta-analysis results in a fundamental change, the meta-analysis results are less stable and reliable.

#### 2.6.5 Publication bias

In the meta-analysis, publication bias is a very important part that directly affects the validity of the conclusion.^[37]^ If there are 10 or more studies in the network meta-analysis, we will use the funnel plot to evaluate the potential publication bias, the funnel plot can directly reflect whether the effect value of the original study is related to the sample size.^[38]^

### 2.7 Quality of evidence

Two reviewers (MY, YTC) will use the Grading of Recommendations Assessment, Development and Evaluation method to assess the quality of evidence of included studies. The evidence levels classified into 4 levels: high, moderate, low, or very low.

## 3 Discussion

Endometrial cancer is a common gynecologic cancer; the treatment for EC is primarily surgical in operable patients, and surgery is usually curative and is the mainstay of initial treatment for most patients with EC, hysterectomy and bilateral salpingo-oophorectomy is usually the gold standard for treatment, but different surgical methods have different advantages and disadvantages. Overall, this network meta-analysis will be the 1st to assess the impact of 5 surgical approaches to EC on patients with cancer; the results of this NMA may provide practical guidance for the clinic and provide new research ideas for researchers.

## Author contributions

**Data curation:** Yitong Cai.

**Methodology:** Yitong Cai.

**Software:** Yitong Cai.

**Writing – original draft:** Li Zhou.

**Writing – review & editing:** Min Yin.
